# Heterogeneity and Differentiation of the Human Arterial Tree: Focus on microRNA Expression in Vascular Disease

**DOI:** 10.3390/biom14030343

**Published:** 2024-03-12

**Authors:** Carmen Ciavarella, Ilenia Motta, Miriam Capri, Mauro Gargiulo, Gianandrea Pasquinelli

**Affiliations:** 1 Department of Medical and Surgical Sciences (DIMEC), University of Bologna, 40138 Bologna, Italy; carmen.ciavarella2@unibo.it (C.C.); miriam.capri@unibo.it (M.C.); gianandr.pasquinelli@unibo.it (G.P.); 2 Alma Mater Institute on Healthy Planet, University of Bologna, 40138 Bologna, Italy; 3 Vascular Surgery Unit, IRCCS Azienda Ospedaliero-Universitaria di Bologna, 40138 Bologna, Italy; mauro.gargiulo2@unibo.it; 4 Vascular Surgery, Department of Medical and Surgical Sciences (DIMEC), University of Bologna, 40138 Bologna, Italy; 5 Pathology Unit, IRCCS Azienda Ospedaliero-Universitaria di Bologna, 40138 Bologna, Italy

**Keywords:** arterial tree, arterial heterogeneity, microRNA, vascular stem cells, differentiation

## Abstract

Human arteries show structural and functional peculiarities according to the nutrient and oxygen needs of a specific vascular district. This architectural heterogeneity is reflected in the pathological setting of cardiovascular diseases (CVDs). Indeed, the responsiveness to cardiovascular risk factors, and the morphological and molecular patterns are discriminating factors among CVDs affecting different vascular beds. MicroRNAs (miRNAs) are endogenous regulators of gene expression and fine-tuners of vascular cell differentiation; thus, these non-coding RNAs can modulate arterial heterogeneity. The identification of an artery-specific miRNA signature would be promising in the therapy of CVDs, especially in patients who are frail and elderly. In the present review, we will provide a concise description of the arterial tree heterogeneity on a structural and cellular basis, mainly in the pathological context. Secondly, we will address the miRNA potential as crucial mediators of arterial heterogeneity, focusing on the abdominal aorta and femoral artery, with the final goal of strengthening the search for more targeted therapies in CVDs and stratification approaches in patients who are frail and elderly.

## 1. Introduction

Arteries constitute a critical part of the cardiovascular system, being the blood vessels that supply the whole body with oxygen and nutrients. Structural and functional features characterize the vascular beds of different anatomical locations within the arterial network. This arterial heterogeneity exerts a significant influence on cardiovascular diseases (CVDs), prompting the search for therapies and surgical options specific to the injured vascular bed.

In the present review, we will discuss arterial tree heterogeneity on a structural and cellular basis, focusing on the differences in atherosclerotic plaques among different arteries. Secondly, the role of microRNA (miRNAs) as potential epigenetic regulators of arterial heterogeneity and differentiation will be proposed, with particular emphasis on the abdominal aorta and femoral artery.

## 2. The Heterogeneity of the Arterial Tree: Structural and Cellular Basis

The cardiovascular system consists of the heart and blood vessels, such as the arteries, veins, and capillaries. Arteries nourish tissue and organs by delivering blood with oxygen and nutrients from the heart to the whole body and are classified into elastic, muscular, and arterioles according to their size and structure. The complexity and heterogeneity of the vascular beds within the arterial tree depend on the different local demands for oxygen and nutrients. The aorta and carotid artery belong to the category of elastic arteries that collect blood from the heart, characterized by a large content of elastic fibers in the tunica media. These fibers allow the vessel to stretch during systole and contract during diastole, facilitating blood distribution within the cardiovascular system [1]. Muscular arteries include the brachial, radial, and femoral arteries. They are medium-sized, collect and deliver blood from elastic arteries to organs, and are equipped with strong connective tissue and less elastic fibers than elastic arteries [1]. Arterioles lead blood into the capillaries, are smaller in size and wall thickness, have a prominent elastic internal membrane, and regulate pressure and blood flow [2]. Their vascular heterogeneity also stems from their vascular cell features and transcriptome. Endothelial cells (ECs) represent the inner cell layer within blood vessels and regulate a broad range of processes, including vascular homeostasis, blood flow regulation, blood cell luminal adherence, and vascular permeability. Despite a shared structure and gene signature, phenotypic and functional heterogeneity emerge in the endothelia from different vascular beds, mirroring differential antigen and transcriptional patterns. Endothelium heterogeneity can be related to embryological origin [3,4,5], the microenvironment [5,6], epigenetics [5,7], site-specific demands, and artery size. In vivo [8,9,10] and in vitro [11] studies support the anatomical differences among ECs. Site-specific transcriptional signatures were observed in bovine endothelial cells derived from their glomerular and aortic endothelia [8]. A microarray analysis also demonstrated a different transcriptome existing between porcine endothelial cells from their coronary and iliac arteries [9]. Differences in terms of growth rate and biochemical properties were also found in ECs isolated from human cerebral and peripheral muscular arteries [11].

Smooth muscle cells (SMCs) are located within the media layer of the arterial wall, regulate the luminal diameter via contraction and relaxation to adapt to the blood flow and to maintain blood pressure, and synthesize many components of the extracellular matrix (ECM). SMCs display a significant plasticity, by switching from a quiescent contractile phenotype to a synthetic proliferative one [12,13]. Beyond phenotypic plasticity, SMC populations arising from different progenitors can be found within the arterial tree, contributing to vascular heterogeneity [13,14]. SMC heterogeneity was also observed in different areas of the same vessel, as documented by the different expressions of desmin and connexin 43 in the SMCs of the internal thoracic artery [15,16].

Vascular stem cells critically affect arterial differentiation concerning structural/functional heterogeneity and disease-pathogenic features. A broad range of evidence supports the existence of a vasculogenic niche, which is an adventitial reservoir of stem cells arising from bone marrow, circulation, and large and small blood vessels [17]. A noteworthy contribution is exerted by the adventitial mesenchymal stromal cells (MSCs), multipotent stem cells with the potential to differentiate into the adipogenic, osteogenic, chondrogenic, and leyomiogenic lineages [18,19]. Functional peculiarities regarding the differentiation potential among MSCs located in different anatomical sites of the arterial tree also exist, as elucidated by comparative studies by our group. Thoracic aorta hMSCs were shown to be more osteogenic than femoral artery ones, whereas the latter displayed a higher propensity to differentiate into the adipogenic lineage according to higher lipid droplet production and peroxisome proliferator-activated receptor (*PPAR*)-γ expression [20]. The chondrogenic potential was more pronounced in hMSCs from the brachiocephalic artery and thoracic aorta than in the hMSCs of the femoral artery [21].

**Figure 1:**
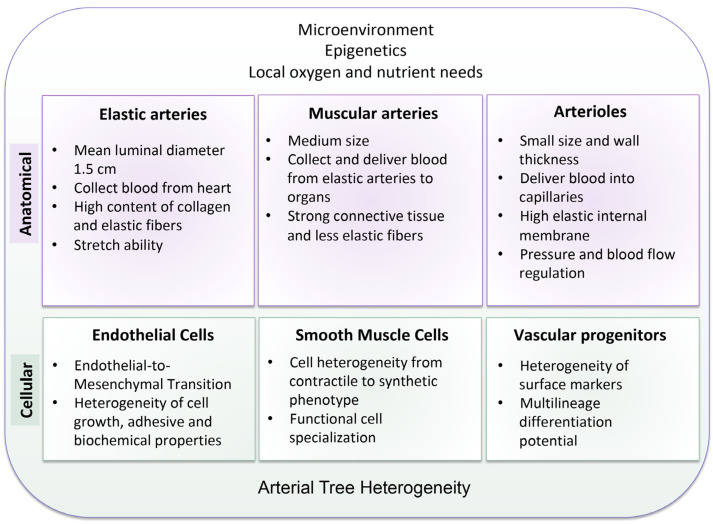
Arterial tree heterogeneity. The two main levels of arterial tree heterogeneity are the anatomical and the cellular ones. The microenvironment, epigenetic regulation, and the different oxygen and nutrient needs according to the anatomical site shape the heterogeneity among different arterial beds.

A graphical scheme illustrating the main levels and contributing factors involved in arterial tree heterogeneity is displayed in Figure 1.

This functional heterogeneity mirrors the different pathological patterns and progressions of CVDs, supporting the higher prevalence of obstructive diseases in femoral vascular beds and the common occurrence of calcified plaques within carotid and thoracic segments, where the degree of osteo-chondrogenic differentiation is higher [21].

The identification of an artery-specific genetic signature represents the key point in cardiovascular research in light of more effective therapies and surgical options for CVDs.

## 3. The Arterial Heterogeneity in Atherosclerosis

Atherosclerosis is the main cause of CVDs, including myocardial infarction, stroke, and heart failure. This chronic and inflammatory affection determines the accumulation of lipids, inflammatory cells, and SMCs, leading to the intimal and medial thickening of medium-sized arteries, progressively reducing the vascular lumen [22]. Smoking, a lipid-rich diet, hypertension, obesity, and high glucose levels are the most common risk factors in atherosclerosis; however, they do not affect all the arterial segments in the same manner. Differences in atherosclerotic plaque pathogenesis, morphology, and progression exist along the arterial tree. Further, not all arterial segments are vulnerable atherosclerotic sites, like the internal mammary artery. The study of atherosclerosis and CVD heterogeneity within the arterial tree would be pivotal for improvements in the clinical and therapeutic options.

Endothelial dysfunction is the initiating event of atherosclerosis, allowing for the recruitment of monocytes, the infiltration of low-density lipoproteins (LDLs) within the intima, and their oxidation. Inflammation, SMC proliferation, and the activation of matrix-degrading enzymes sharpen the pathological setting. Under these conditions, secondary mechanisms may occur, like ectopic calcification, which is calcium deposition in the vascular wall associated with plaque instability, rupture, and an increase in mortality risk [23]. A broad range of evidence supports an anatomical specificity for atherosclerosis and heterogeneity in the morphological and pathogenic mechanisms due to differences in crucial factors like hemodynamic, wall architecture, and vascular cell characteristics (i.e., SMC developmental origin) among different arteries [24,25]. Morphological and hemodynamic differences can be seen in carotid and coronary plaques; indeed, carotid plaques exhibit thicker fibrous caps, a higher prevalence of intraplaque hemorrhage, and calcification compared to coronary plaques [26].

A morphological study based on the American Heart Association (AHA) grading, calcification, and lipid content highlighted differences between the plaques of carotid and femoral arteries. Carotid plaques were mainly characterized by a fibrous cap atheroma, whereas fibrocalcific plaques were mainly observed in femoral arteries; further, femoral plaques displayed higher calcium and lower cholesterol contents than carotid plaques [24]. The relevance of vascular cell heterogeneity has been elucidated in a histological and transcriptomic study assessing healthy and atherosclerotic plaques from different vascular beds. In this study, femoral plaques resulted in the most calcified arteries and the transcriptomic profile of SMCs was consistent with a prominent mineralization activity, whereas SMCs from other arterial segments, like abdominal and thoracic aortas, were less prone to calcification [27]. As introduced above, developmental origin and epigenetics are some of the mechanisms proposed to be responsible for vascular cell heterogeneity. Epigenetics refers to the modulation of gene expression through histone modifications, DNA methylation, and noncoding RNAs (ncRNAs), without involving DNA structure [28]. ncRNAs include microRNAs (miRNAs), a class of small non-coding endogenous RNAs (10–22 nucleotides) that regulate the expression of different genes involved in many biological processes. The first miRNA was discovered in 1993 by Victor Ambros, Rosalind Lee, and Rhonda Feinbaum while studying the gene lin-4, which controls the timing of *C. elegans* larval development [29]. Considering the key regulatory role of miRNAs in vascular cell phenotype and function, it could be reasonable to hypothesize a miRNA-based molecular signature of arterial beds useful for deepening atherosclerosis and CVD pathogenesis differentiation and for developing more targeted and effective preventive/therapeutic interventions. In the management of CVDs, the identification of patients who are frail and at higher mortality risk should be included. Frailty refers to an age-related clinical syndrome with multiple organ affections including CVDs, and higher vulnerability to negative health outcomes [30,31]. Age is a well-known risk factor, but genetic, epigenetic, and environmental stressors are also potent stressors in frail conditions. However, frailty has not been fully defined yet and the identification of biomarkers able to screen patients who are frail would be promising in CVD management.

## 4. microRNAs (miRNAs)

### 4.1. miRNAs as Endogenous Regulators of Vascular Biology and Vascular Heterogeneity

miRNAs regulate specific cellular processes through the inhibition of the expression of their gene targets by inducing messenger RNA (mRNA) degradation or inhibiting mRNA translation, resulting in the inhibition of protein synthesis [32].

**Figure 2:**
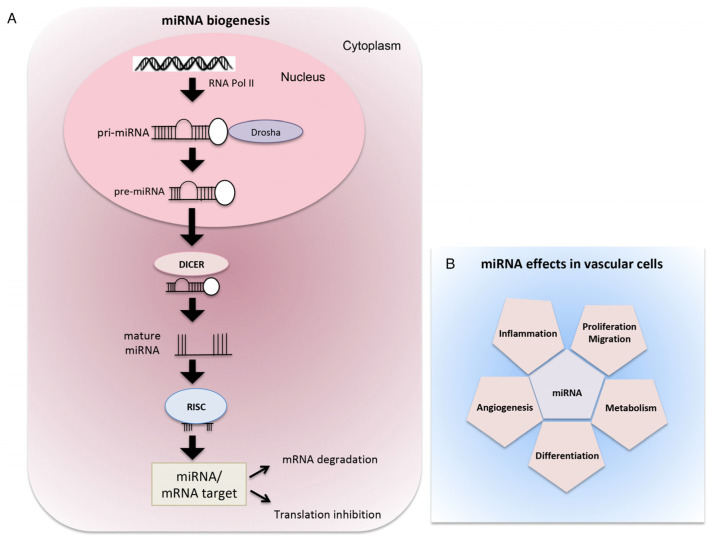
The canonical biogenesis and effects of miRNA in vascular cells. (**A**) miRNAs are synthesized in the nucleus by RNA polymerase II into pri-miRNA transcripts, which in turn are cleaved by RNase III enzyme Drosha into the pre-miRNA precursor. After, pre-miRNA translocates to the cytoplasm where the complex with RNase III Dicer processes the precursor into the mature miRNA. The mature miRNA binds to the target mRNA in correspondence with the complementary sites and regulates gene expression by degrading mRNA or by suppressing the translation process. (**B**) In vascular cells, miRNAs orchestrate a broad range of biological processes associated with vascular homeostasis and pathology: cell proliferation, differentiation, migration, metabolism, angiogenesis, and inflammation.

The canonical biogenesis of miRNAs begins from their precursors, called primary miRNA transcripts (pri-miRNAs), in the nucleus, where they are cleaved by the ribonuclease DROSHA into shorter temporary miRNAs (pre-miRNAs). Pre-miRNAs are then transported from the nucleus to the cytoplasm and processed by another ribonuclease, DICER, into short double-stranded immature miRNAs. Subsequently, one strand is degraded by the component 3 promoter of the RNA-induced silencing complex (C3PO), whereas the guide strand is loaded into the RNA-induced silencing complex (RISC). Here, miRNAs can bind to their mRNA targets and inhibit their expression through the endonuclease cleavage activity of the protein Argonaute 2 (AGO2) [33] (Figure 2).

miRNAs contribute to the regulation of both coding and non-coding RNA transcriptomes in the nucleus by blocking or promoting pri-miRNA maturation and by regulating long non-coding RNAs levels. Moreover, in the nucleus, miRNAs induce the remodeling of chromatin structures; regulate alternative splicing; and in turn, regulate itself. miRNAs can also mediate transcriptional gene activation or transcriptional gene silencing. Other nuclear miRNAs co-localize with ribosomal RNAs (rRNAs) in the nucleolus, where they are stored, or in the cytoplasm, where they influence the abundance of rRNAs and/or regulate ribosome interaction with accessory proteins. All these miRNA activities have recently been reviewed [34].

A large number of miRNAs are involved in different biological and pathological processes such as cell proliferation, differentiation, and migration [35,36]; inflammation [37,38]; nervous system diseases [39,40,41]; cancer [42]; and diabetes [43].

In the cardiovascular system, miRNAs regulate the development process as well as the pathogenesis of many diseases (Figure 2).

Fish et al. observed that miR-126 is highly expressed in endothelial cells and it is important for angiogenesis and for the maintenance of vascular integrity in vivo as it stimulates vascular endothelial growth factor (*VEGF*) signaling by directly inhibiting *SPRED1* and *PIK3R2*, which are negative regulators of VEGF signaling [44]. Moreover, miR-23 and miR-27 are positive regulators of angiogenesis in vivo, inhibiting Sprouty2 and semaphoring 6A (*SEMA6A*), which negatively regulate Mapk and Vegf2 signaling [45,46]. In endothelial cells, miR-210 overexpression led to the upregulation of the *NOTCH1* pathway, which is responsible for the enhanced blood vessel formation of the endothelium [47]. In vascular SMCs, one of the most expressed miRNA is miR-15b/16, whose overexpression promotes the contractility of VSMCs while mitigating their proliferation through the inhibition of the oncoprotein Yes-associated protein (*YAP*) [48]. Also, it has been found that the levels of miR-146 and miR-31 expression are higher in proliferative VSMCs and silencing these miRNAs results in the inhibition of VMSCs’ proliferative and migratory abilities, thus demonstrating that they have pro-proliferative and anti-apoptotic functions [49,50].

The dysregulation of miRNA expression can lead to several pathological processes, including the pathogenesis of atherosclerosis [51]. The upregulation of mir-21, miR-92a, and miR-was found in atherosclerotic plaques; in particular, miR-21 was higher in symptomatic carotid plaques in comparison to asymptomatic ones [52]. Zhou et al. quantified the serum expression levels of the miR-30-5p family in patients with atherosclerosis compared to a normal group, observing that the expression levels of miR-30-5p were higher in the atherosclerosis group and supporting the miR-30a-5p association with cardiovascular disease [53]. Moreover, a study on atherosclerotic plaques showed that miR-30a-5p and miR-30d were downregulated in calcified carotid plaques, identifying them as potential contributors to vascular calcification [54]. Indeed, in an in vitro study in human umbilical vein endothelial cells (HUVECs), miR-30a-5p and miR-30d became downregulated following osteogenic differentiation, and their over-expression during the osteogenic induction assay revealed a decrease in mineralization activity, implying that these miRNAs potentially exert a regulatory role in atherosclerotic calcification [55]. In addition, Han et al. demonstrated that miR-223-3p expression increases in medial and atherosclerotic calcified aortas while suppressing vascular calcification by targeting *IL-6/STAT3* signaling [56]. Further mechanisms that involve miRNAs in vascular cell regulation are reported in a recent systematic review [57]. The main findings reported in this section are summarized in Table 1. miRNA fluctuations during the disease progression suggest that some miRNAs can be stage-specific, as elucidated in a microarray analysis of early and advanced stages of atherosclerotic plaques [58]. In that study, the authors identified two significant miRNA/mRNA signatures unique for early and advanced stages, finding correspondence also in peripheral blood [58].

The miRNA contribution to vascular disease dynamics is very complex and versatile; deepening their role and mechanisms would pave the way for miRNAs to be used as markers for diagnostic purposes and as a potential foundation for therapeutic strategies.

### 4.2. miRNAs and Vascular Heterogeneity: Focus on Femoral Artery and Abdominal Aorta

miRNAs are fine-tuners of cell biology and phenotype, suggesting their contribution to vascular heterogeneity. So far, there are few studies addressing the diversity of miRNA expression in normal tissues. However, the presence of many organ-specific miRNAs has been observed in mice [63], but the possible differences among cells belonging to the same tissue have not yet been elucidated. A recent profiling study focused also on pri-miRNAs and highlighted that these primary miRNAs are cell-type specific but less susceptible to pro-atherogenic stimuli; conversely, mature miRNAs were found to be shared quite often among cells of endothelial, smooth muscle, and inflammatory (macrophage) origin [64]. A comparative study identified three miRNAs (miR-20b, miR-99b, and let-7b) that were differently expressed among ECs cultured from aorta, coronary artery, umbilical vein, pulmonary, dermal, and brain microvasculature [65]. The literature is also poor regarding comparative studies based on miRNA expression profiles in different arterial districts under pathological conditions. A systematic review found both a common and a site-specific profile of circulating miRNAs in individuals with and without atherosclerosis of large or medium size [66]. Some miRNAs displayed opposite expression trends, like miR-126, which was upregulated in renal artery stenosis and downregulated in carotid and lower limb plaques [66]. Conversely, miR-221-3p/miR-30 downregulation and miR-21 upregulation were found in both carotid and lower limbs arteries, whereas miR-145 was downregulated in carotid and coronary arteries [67].

The recent knowledge acquired on miRNA genome regulation further supports the hypothesis of their central contribution to arterial structure differences and atherosclerosis development. In this regard, Collura et al. [68] showed that miR profiling in non-pathological femoral, abdominal, and carotid arteries presents high similarity between the abdominal aorta and carotid arteries, while a major difference is observed between the femoral artery and the other two arteries. Those authors identified that three miRNAs were significantly altered compared with normal arteries, i.e., miR-27a-5p, -139-5p, and -155-5p. In particular, miR-155-5p and miR-27a-5p turned out to be more highly expressed in normal aorta/carotids than femoral arteries, while miR-139-5p showed an opposite trend, suggesting a different epigenetic pattern in the normal arteries. These three miRNAs also have relevance under pathological conditions. In fact, the same authors showed that miR-155-5p and miR-27a-5p expression increases in femoral atheroma when compared with the normal counterpart, thus becoming more like normal abdominal/carotid aorta arteries. Furthermore, some targets of the identified miRNAs, such as CD44, E-cadherin, and vimentin, were shown to be differently expressed under physiological and disease conditions between femoral and abdominal/carotid arteries, also suggesting that in these arteries, there is a different type of activation of the main molecular drivers of pathological condition. This current topic is almost neglected and deserves additional research that should focus on blood/vesicle-circulating miRNAs to investigate their effects on atheroma development in femoral and abdominal aorta arteries. Interestingly, in obese animal models, extracellular vesicles with miR-221-3p derived from perivascular adipose tissue mediate vascular remodeling and dysfunction in the femoral artery [69]. In this regard, further studies are crucial to identifying both common and different epigenetic molecular patterns to pave the way for future artery-specific therapeutic applications [70].

## 5. Conclusions

The complex nature of the arterial network is reflected in the physiological functionality of vascular tissues and in the pathogenic mechanisms affecting vascular beds according to their anatomical localization. An investigation of this heterogeneity, including the different responsiveness to the main pathology risk factors and the several pathways activated during disease occurrence, would be key to more effective and targeted therapies for the management of CVDs. Among the pathogenic mechanisms, vascular stem cell differentiation performs a pivotal role in CVD progression, contributing to the main morphological features of CVDs from different arterial segments. The identification of a miRNA signature that can be associated with the differentiation process and is specific for each vascular district would open novel research directions for the future of artery-specific approaches. In this future perspective, blood-circulating miRNAs can be employed as diagnostic tools for risk stratification also in the elderly population [71] to identify patients at greater risk of cardiovascular mortality.

## Figures and Tables

**Table 1:** miRNAs involved in the regulation of vascular tissue physiology and disease.

miRNA	Roles in the Vascular System
miR-126	Regulation of angiogenic process and maintenance of vascular integrity by activating VEGF signaling [41]
miR-23, miR-27	Promotion of angiogenic process by inhibition of *SPROUTY2* and *SEMA6A* [42,43]
miR-210	Induction of *NOTCH1* pathway [44]
miR-15b/16	Promotion of VSMC contractile phenotype [45]
miR-146, miR-31	Control of proliferative and migratory abilities in VSMCs [46,47]
miR-30a-5p, miR-30d	Regulation of End-MT, osteogenic differentiation, and vascular calcification by targeting SLUG [51]
miR-223-3p	Inhibition of vascular calcification by targeting *IL-6/STAT3* signaling [56]
miR-155-5p, miR-27a-5p	Increased expression in atherosclerotic plaque of femoral arteries [54]
miR-183-5p, miR-488	Increase of proliferation and migration in VSMCs [59,60]
miR-378a	Inhibition of proliferation and migration in VSMCs [61]
miR-217	Promotion of endothelial dysfunction [62]

miRNA: microRNA; VEGF: vascular endothelial growth factor; SEMA6A: semaphorin 6A; Notch-1: neurogenic locus notch homolog protein 1; VSMC: vascular smooth muscle cells; End-MT: endothelial–mesenchymal transition; SLUG: snail family transcription repressor 2; IL-6: interleukin-6; STAT3: signal transducer and activator of transcription 3.

## References

[1] Tucker W.D., Arora Y., Mahajan K. (2022). Anatomy, Blood Vessels. StatPearls.

[2] Fleischer J.R., Jodszuweit C.A., Ghadimi M., De Oliveira T., Conradi L.-C. (2020). Vascular Heterogeneity With a Special Focus on the Hepatic Microenvironment. Front. Physiol..

[3] Aird W.C. (2006). Endothelial Cell Heterogeneity and Atherosclerosis. Curr. Atheroscler. Rep..

[4] Aird W.C. (2007). Phenotypic Heterogeneity of the Endothelium. Circ. Res..

[5] Aird W.C. (2012). Endothelial Cell Heterogeneity. Cold Spring Harb. Perspect. Med..

[6] Potente M., Mäkinen T. (2017). Vascular Heterogeneity and Specialization in Development and Disease. Nat. Rev. Mol. Cell Biol..

[7] Chi J.-T., Chang H.Y., Haraldsen G., Jahnsen F.L., Troyanskaya O.G., Chang D.S., Wang Z., Rockson S.G., van de Rijn M., Botstein D. (2003). Endothelial Cell Diversity Revealed by Global Expression Profiling. Proc. Natl. Acad. Sci. USA.

[8] Sengoelge G., Luo W., Fine D., Perschl A.M., Fierlbeck W., Haririan A., Sorensson J., Rehman T.-U., Hauser P., Trevick J.S. (2005). A SAGE-Based Comparison between Glomerular and Aortic Endothelial Cells. Am. J. Physiol.-Ren. Physiol..

[9] Zhang J., Burridge K.A., Friedman M.H. (2008). In Vivo Differences between Endothelial Transcriptional Profiles of Coronary and Iliac Arteries Revealed by Microarray Analysis. Am. J. Physiol. Heart Circ. Physiol..

[10] Simmons G.H., Padilla J., Laughlin M.H. (2012). Heterogeneity of Endothelial Cell Phenotype within and amongst Conduit Vessels of the Swine Vasculature. Exp. Physiol..

[11] Thorin E., Shatos M.A., Shreeve S.M., Walters C.L., Bevan J.A. (1997). Human Vascular Endothelium Heterogeneity. Stroke.

[12] Rensen S.S.M., Doevendans P.A.F.M., van Eys G.J.J.M. (2007). Regulation and Characteristics of Vascular Smooth Muscle Cell Phenotypic Diversity. Neth. Heart J..

[13] Wall V.Z., Bornfeldt K.E. (2014). Arterial Smooth Muscle. Arterioscler. Thromb. Vasc. Biol..

[14] Majesky M.W. (2007). Developmental Basis of Vascular Smooth Muscle Diversity. Arterioscler. Thromb. Vasc. Biol..

[15] Ko Y.-S., Yeh H.-I., Haw M., Dupont E., Kaba R., Plenz G., Robenek H., Severs N.J. (1999). Differential Expression of Connexin43 and Desmin Defines Two Subpopulations of Medial Smooth Muscle Cells in the Human Internal Mammary Artery. Arterioscler. Thromb. Vasc. Biol..

[16] Li S., Fan Y.-S., Chow L.H., Van Den Diepstraten C., van der Veer E., Sims S.M., Pickering J.G. (2001). Innate Diversity of Adult Human Arterial Smooth Muscle Cells. Circ. Res..

[17] Ciavarella C., Valente S., Pasquinelli G. (2022). The Characteristics and Survival Potential Under Sub-Lethal Stress of Mesenchymal Stromal/Stem Cells Isolated from the Human Vascular Wall. Stem Cells.

[18] Tang Z., Wang A., Yuan F., Yan Z., Liu B., Chu J.S., Helms J.A., Li S. (2012). Differentiation of Multipotent Vascular Stem Cells Contributes to Vascular Diseases. Nat. Commun..

[19] Pasquinelli G., Pacilli A., Alviano F., Foroni L., Ricci F., Valente S., Orrico C., Lanzoni G., Buzzi M., Luigi Tazzari P. (2010). Multidistrict Human Mesenchymal Vascular Cells: Pluripotency and Stemness Characteristics. Cytotherapy.

[20] Pasanisi E., Ciavarella C., Valente S., Ricci F., Pasquinelli G. (2019). Differentiation and Plasticity of Human Vascular Wall Mesenchymal Stem Cells, Dermal Fibroblasts and Myofibroblasts: A Critical Comparison Including Ultrastructural Evaluation of Osteogenic Potential. Ultrastruct. Pathol..

[21] Sabrina V., Gianandrea P. (2017). Phenotypic and Functional Mapping of Mesenchymal Stem Cells Harvested from Different Portions of the Human Arterial Tree. Mesenchymal Stem Cells—Isolation, Characterization and Applications.

[22] Lahoz C., Mostaza J.M. (2007). Atherosclerosis As a Systemic Disease. Rev. Esp. Cardiol..

[23] Stary H.C., Chandler A.B., Dinsmore R.E., Fuster V., Glagov S., Insull W., Rosenfeld M.E., Schwartz C.J., Wagner W.D., Wissler R.W. (1995). A Definition of Advanced Types of Atherosclerotic Lesions and a Histological Classification of Atherosclerosis. Circulation.

[24] Herisson F., Heymann M.-F., Chétiveaux M., Charrier C., Battaglia S., Pilet P., Rouillon T., Krempf M., Lemarchand P., Heymann D. (2011). Carotid and Femoral Atherosclerotic Plaques Show Different Morphology. Atherosclerosis.

[25] Artery-Related Differences in Atherosclerosis Expression|Stroke. https://www.ahajournals.org/doi/10.1161/STROKEAHA.107.486480.

[26] Sigala F., Oikonomou E., Antonopoulos A.S., Galyfos G., Tousoulis D. (2018). Coronary versus Carotid Artery Plaques. Similarities Differ. Regarding Biomark. Morphol. Prognosis. Curr. Opin. Pharmacol..

[27] Espitia O., Chatelais M., Steenman M., Charrier C., Maurel B., Georges S., Houlgatte R., Verrecchia F., Ory B., Lamoureux F. (2018). Implication of Molecular Vascular Smooth Muscle Cell Heterogeneity among Arterial Beds in Arterial Calcification. PLoS ONE.

[28] Aavik E., Babu M., Ylä-Herttuala S. (2019). DNA Methylation Processes in Atherosclerotic Plaque. Atherosclerosis.

[29] Lee R.C., Feinbaum R.L., Ambros V. (1993). The *C. Elegans* Heterochronic Gene Lin-4 Encodes Small RNAs with Antisense Complementarity to Lin-14. Cell.

[30] Singh M., Stewart R., White H. (2014). Importance of Frailty in Patients with Cardiovascular Disease. Eur. Heart J..

[31] Dato S., Crocco P., Iannone F., Passarino G., Rose G. (2022). Biomarkers of Frailty: miRNAs as Common Signatures of Impairment in Cognitive and Physical Domains. Biology.

[32] Small E.M., Olson E.N. (2011). Pervasive Roles of microRNAs in Cardiovascular Biology. Nature.

[33] Stavast C.J., Erkeland S.J. (2019). The Non-Canonical Aspects of MicroRNAs: Many Roads to Gene Regulation. Cells.

[34] Annese T., Tamma R., De Giorgis M., Ribatti D. (2020). microRNAs Biogenesis, Functions and Role in Tumor Angiogenesis. Front. Oncol..

[35] Wang D., Atanasov A.G. (2019). The microRNAs Regulating Vascular Smooth Muscle Cell Proliferation: A Minireview. Int. J. Mol. Sci..

[36] Kim D.Y., Sung J.H. (2016). Regulatory Role of microRNAs in the Proliferation and Differentiation of Adipose-Derived Stem Cells. Histol. Histopathol..

[37] miR-21 and miR-146a: The microRNAs of Inflammaging and Age-Related Diseases—ScienceDirect. https://www.sciencedirect.com/science/article/pii/S1568163721001215?via%3Dihub.

[38] Mahesh G., Biswas R. (2019). MicroRNA-155: A Master Regulator of Inflammation. J. Interferon Cytokine Res..

[39] microRNAs at the Synapse|Nature Reviews Neuroscience. https://www.nature.com/articles/nrn2763.

[40] Zheng K., Li H., Huang H., Qiu M. (2012). microRNAs and Glial Cell Development. Neuroscientist.

[41] Zingale V.D., Gugliandolo A., Mazzon E. (2021). MiR-155: An Important Regulator of Neuroinflammation. Int. J. Mol. Sci..

[42] Lee Y.S., Dutta A. (2009). MicroRNAs in Cancer. Annu. Rev. Pathol. Mech. Dis..

[43] Tang X., Tang G., Özcan S. (2008). Role of MicroRNAs in Diabetes. Biochim. Biophys. Acta.

[44] Fish J.E., Santoro M.M., Morton S.U., Yu S., Yeh R.-F., Wythe J.D., Bruneau B.G., Stainier D.Y.R., Srivastava D. (2008). miR-126 Regulates Angiogenic Signaling and Vascular Integrity. Dev. Cell.

[45] Zhou Q., Gallagher R., Ufret-Vincenty R., Li X., Olson E.N., Wang S. (2011). Regulation of Angiogenesis and Choroidal Neovascularization by Members of microRNA-23∼27∼24 Clusters. Proc. Natl. Acad. Sci. USA.

[46] Urbich C., Kaluza D., Frömel T., Knau A., Bennewitz K., Boon R.A., Bonauer A., Doebele C., Boeckel J.-N., Hergenreider E. (2012). MicroRNA-27a/b Controls Endothelial Cell Repulsion and Angiogenesis by Targeting Semaphorin 6A. Blood.

[47] Lou Y.-L., Guo F., Liu F., Gao F.-L., Zhang P.-Q., Niu X., Guo S.-C., Yin J.-H., Wang Y., Deng Z.-F. (2012). miR-210 Activates Notch Signaling Pathway in Angiogenesis Induced by Cerebral Ischemia. Mol. Cell Biochem..

[48] Xu F., Ahmed A.S.I., Kang X., Hu G., Liu F., Zhang W., Zhou J. (2015). MicroRNA-15b/16 Attenuates Vascular Neointima Formation by Promoting the Contractile Phenotype of Vascular Smooth Muscle through Targeting YAP. Arterioscler. Thromb. Vasc. Biol..

[49] Liu X., Cheng Y., Chen X., Yang J., Xu L., Zhang C. (2011). MicroRNA-31 Regulated by the Extracellular Regulated Kinase Is Involved in Vascular Smooth Muscle Cell Growth via Large Tumor Suppressor Homolog 2. J. Biol. Chem..

[50] Dong S., Xiong W., Yuan J., Li J., Liu J., Xu X. (2013). MiRNA-146a Regulates the Maturation and Differentiation of Vascular Smooth Muscle Cells by Targeting NF-κB Expression. Mol. Med. Rep..

[51] Vartak T., Kumaresan S., Brennan E. (2022). Decoding microRNA Drivers in Atherosclerosis. Biosci. Rep..

[52] Parahuleva M.S., Lipps C., Parviz B., Hölschermann H., Schieffer B., Schulz R., Euler G. (2018). MicroRNA Expression Profile of Human Advanced Coronary Atherosclerotic Plaques. Sci. Rep..

[53] Zhou Z., Chen Y., Zhang D., Wu S., Liu T., Cai G., Qin S. (2019). MicroRNA-30-3p Suppresses Inflammatory Factor-Induced Endothelial Cell Injury by Targeting TCF21. Mediat. Inflamm..

[54] Vasuri F., Ciavarella C., Fittipaldi S., Pini R., Vacirca A., Gargiulo M., Faggioli G., Pasquinelli G. (2019). Different Histological Types of Active Intraplaque Calcification Underlie Alternative miRNA-mRNA Axes in Carotid Atherosclerotic Disease. Virchows Arch..

[55] Ciavarella C., Motta I., Vasuri F., Fittipaldi S., Valente S., Pollutri D., Ricci F., Gargiulo M., Pasquinelli G. (2021). Involvement of miR-30a-5p and miR-30d in Endothelial to Mesenchymal Transition and Early Osteogenic Commitment under Inflammatory Stress in HUVEC. Biomolecules.

[56] Han Y., Zhang J., Huang S., Cheng N., Zhang C., Li Y., Wang X., Liu J., You B., Du J. (2021). MicroRNA-223-3p Inhibits Vascular Calcification and the Osteogenic Switch of Vascular Smooth Muscle Cells. J. Biol. Chem..

[57] Li Z., Zhao Y., Suguro S., Suguro R. (2023). MicroRNAs Regulate Function in Atherosclerosis and Clinical Implications. Oxid. Med. Cell Longev..

[58] Verma S., Kumar A., Narang R., Bisoi A.K., Mitra D.K. (2022). Signature Transcriptome Analysis of Stage Specific Atherosclerotic Plaques of Patients. BMC Med. Genom..

[59] Sun B., Shan Z., Sun G., Wang X. (2021). Micro-RNA-183-5p Acts as a Potential Diagnostic Biomarker for Atherosclerosis and Regulates the Growth of Vascular Smooth Muscle Cell. J. Chin. Med. Assoc..

[60] Li Z., Xu C., Sun D. (2021). MicroRNA-488 Serves as a Diagnostic Marker for Atherosclerosis and Regulates the Biological Behavior of Vascular Smooth Muscle Cells. Bioengineered.

[61] Chong H., Wei Z., Na M., Sun G., Zheng S., Zhu X., Xue Y., Zhou Q., Guo S., Xu J. (2020). The PGC-1α/NRF1/miR-378a Axis Protects Vascular Smooth Muscle Cells from FFA-Induced Proliferation, Migration and Inflammation in Atherosclerosis. Atherosclerosis.

[62] de Yébenes V.G., Briones A.M., Martos-Folgado I., Mur S.M., Oller J., Bilal F., González-Amor M., Méndez-Barbero N., Silla-Castro J.C., Were F. (2020). Aging-Associated miR-217 Aggravates Atherosclerosis and Promotes Cardiovascular Dysfunction. Arterioscler. Thromb. Vasc. Biol..

[63] Lagos-Quintana M., Rauhut R., Yalcin A., Meyer J., Lendeckel W., Tuschl T. (2002). Identification of Tissue-Specific MicroRNAs from Mouse. Curr. Biol..

[64] Moreau P.R., Tomas Bosch V., Bouvy-Liivrand M., Õunap K., Örd T., Pulkkinen H.H., Pölönen P., Heinäniemi M., Ylä-Herttuala S., Laakkonen J.P. (2021). Profiling of Primary and Mature miRNA Expression in Atherosclerosis-Associated Cell Types. Arterioscler. Thromb. Vasc. Biol..

[65] McCall M.N., Kent O.A., Yu J., Fox-Talbot K., Zaiman A.L., Halushka M.K. (2011). MicroRNA Profiling of Diverse Endothelial Cell Types. BMC Med. Genom..

[66] Pereira-da-Silva T., Coutinho Cruz M., Carrusca C., Cruz Ferreira R., Napoleão P., Mota Carmo M. (2018). Circulating microRNA Profiles in Different Arterial Territories of Stable Atherosclerotic Disease: A Systematic Review. Am. J. Cardiovasc. Dis..

[67] Teixeira A.R., Ferreira V.V., Pereira-da-Silva T., Ferreira R.C. (2022). The Role of miRNAs in the Diagnosis of Stable Atherosclerosis of Different Arterial Territories: A Critical Review. Front. Cardiovasc. Med..

[68] Collura S., Ciavarella C., Morsiani C., Motta I., Valente S., Gallitto E., Abualhin M., Pini R., Vasuri F., Franceschi C. (2021). MicroRNA Profiles of Human Peripheral Arteries and Abdominal Aorta in Normal Conditions: MicroRNAs-27a-5p, -139-5p and -155-5p Emerge and in Atheroma Too. Mech. Ageing Dev..

[69] Li X., Ballantyne L.L., Yu Y., Funk C.D. (2019). Perivascular Adipose Tissue–Derived Extracellular Vesicle miR-221-3p Mediates Vascular Remodeling. FASEB J..

[70] Collura S., Morsiani C., Vacirca A., Fronterrè S., Ciavarella C., Vasuri F., D’Errico A., Franceschi C., Pasquinelli G., Gargiulo M. (2020). The Carotid Plaque as Paradigmatic Case of Site-Specific Acceleration of Aging Process: The microRNAs and the Inflammaging Contribution. Ageing Res. Rev..

[71] Salvioli S., Basile M.S., Bencivenga L., Carrino S., Conte M., Damanti S., De Lorenzo R., Fiorenzato E., Gialluisi A., Ingannato A. (2023). Biomarkers of aging in frailty and age-associated disorders: State of the art and future perspective. Ageing Res. Rev..

